# Assessment of Knowledge, Attitudes, and Practices Regarding Menstrual Hygiene Management Among Adolescent Schoolgirls (10–19 Years) in the Saurashtra Region, Gujarat

**DOI:** 10.7759/cureus.50950

**Published:** 2023-12-22

**Authors:** Yogesh M, Nidhi S Trivedi, Rachita Damor, Monika Patel, Hemangi Ladani, Arya Ramachandran, Roshni Vamja, Bhumika Surati

**Affiliations:** 1 Community Medicine, Shri M. P. Shah Government Medical College, Jamnagar, IND; 2 Obstetrics and Gynaecology, Gujarat Medical Education and Research Society (GMERS) Medical College, Himmatnagar, Himmatnagar, IND; 3 Community and Family Medicine, Guru Gobind Singh Government Hospital, Jamnagar, IND; 4 Preventive Medicine, Shri M. P. Shah Government Medical College, Jamnagar, IND

**Keywords:** practices, adolescents, attitudes, knowledge, menstruation

## Abstract

Background

Menstrual health management proves pivotal for the adoption of hygienic practices and the embracement of womanhood from the onset of menarche. Menstrual hygiene is pivotal yet under-addressed among adolescent girls in India. This study aimed to assess the knowledge, attitudes, and practices regarding menstrual hygiene and influencing factors.

Methodology

A cross-sectional study was conducted among 361 adolescent schoolgirls aged 10-19 years in the Saurashtra region of Gujarat using a pretested questionnaire. Multistage sampling was used. In the first stage, six schools (three rural and three urban) were selected through random sampling. In the second stage, all adolescent girls aged 10-19 years in the selected schools who had attained menarche were invited to participate. Those who provided written assent were included in the study. The data were analyzed with descriptive statistics and logistic regression. A p-value <0.05 was considered significant.

Results

Knowledge about menstruation was evenly distributed as good (47.65%) and poor (52.35%). Most relied on mothers for information and considered menstruation a normal phenomenon. Attitudes reflected complex cultural perceptions. The use of sanitary pads was high (96.12%), although 39.89% used reused absorbents. Multivariate analysis revealed age 16-19 years (adjusted odds ratio (AOR) = 2.08, 95% confidence interval (CI) = 1.14-3.81), higher parental education, pit latrine usage (AOR = 6.7, 95% CI = 2.97-15.15), and knowledge about menstruation (AOR = 8.21, 95% CI = 4.56-14.78) as positive predictors of good menstrual practices.

Conclusions

Despite the widespread use of sanitary pads, the persisting knowledge gap and sociocultural influences lead to unhygienic practices. Improving awareness and attitudes through educational interventions among adolescent girls and mothers, in particular, addressing cultural taboos through the engagement of all stakeholders, and improving sanitation infrastructure in schools are imperative.

## Introduction

The adolescent phase, especially in India, represents a pivotal period for girls, notwithstanding the ceremonial attention given to their “coming of age” [1]. Regrettably, the information disseminated during this period often weaves through a tapestry of myths and taboos, resulting in imposed restrictions and misconceptions surrounding menstruation [2]. Menstrual health management proves pivotal for the adoption of hygienic practices and the embracement of womanhood from the onset of menarche. However, cultural beliefs, such as avoiding bathing, eating specific foods, and exercising, lacking scientific support, contribute to menstrual anxiety among girls [3]. Menstruation, a natural facet of the female reproductive cycle, signifies a crucial developmental milestone that demands attention to menstrual hygiene for the well-being and dignity of women and girls [4]. The practices of menstrual hygiene management (MHM) encompass the use of clean menstrual materials, proper body cleaning with soap and water, and the appropriate disposal of used materials [5]. However, girls’ coping techniques vary widely based on personal choices, available resources, economic status, local customs, cultural values, and education [6]. The global challenge of inadequate access to sanitation services, clean water, and hygiene facilities affects approximately 2.3 billion people, with only 27% of those in developing countries having access to water and soap for handwashing [7]. Compounding this issue is the insufficient infrastructure in schools, with nearly half of low-income nations lacking essential drinking water, sanitation, and hygiene facilities [8]. The repercussions of poor MHM are far-reaching, leading to reproductive and urogenital infections such as pelvic inflammatory disease and dysmenorrhea, as well as infertility. Moreover, the discomfort and stigma associated with menstruation contribute to concentration problems, limited engagement, and reduced confidence among schoolgirls, impacting their academic performance and overall well-being [9]. In Africa, approximately 10% of school-aged girls miss school during menstruation [10], underscoring the imperative for comprehensive interventions. Despite the significance of safe menstrual hygiene practices, many adolescent girls in lower socioeconomic settings lack sufficient knowledge and engage in improper hygiene practices.

The integration of menstrual hygiene promotion into the healthcare system is crucial, necessitating policy implications and extensive efforts to enhance girls’ knowledge and safe hygienic practices regarding menstruation. While studies on menstrual health have been conducted in various parts of the country, our study area in Gujarat remains unexplored. The magnitude of the problem remains unknown, and context-specific social and cultural factors have not been adequately addressed. This study aims to bridge this gap by assessing the knowledge, attitudes, and practices related to MHM among adolescent girls in the Saurashtra region of Gujarat. The findings are anticipated to provide essential clinical insights, inform policy development, and guide tailored intervention strategies. Additionally, the study results will serve as a baseline for future research endeavors.

## Materials and methods

Study design and setting

A cross-sectional study design was adopted to assess the knowledge, attitudes, and practices regarding menstrual hygiene among adolescent schoolgirls in the Saurashtra region of Gujarat during March-September 2023. The study was conducted at government and private girls’ schools across rural and urban areas.

Study participants and sampling

The study population comprised adolescent schoolgirls aged between 10 and 19 years. Multistage sampling was utilized. In the first stage, stratified sampling was done by area, selecting both rural and urban regions. In the second stage, within rural and urban areas, further geographical stratification was done by the north, south, east, and west regions. In the third stage, within each of these sub-regions, three schools were randomly selected. In the fourth stage, from the selected schools in all areas, the study population comprised all adolescent girls aged 10-19 who had attained menarche. These girls were recruited after obtaining assent and through an announcement by school authorities. This two-stage sample frame encompassing the list of schools and rosters of menstruating girls meeting the age criteria allowed for a representative study population to be systematically selected according to the multistage sampling technique delineated. Those who provided written consent were recruited for the study. The minimum required sample size was 361, which was calculated using the formula for estimating a population proportion with absolute precision at a 95% confidence level, assuming a 50% prevalence of good menstrual hygiene practices, and with a 5% margin of error [11].

Inclusion criteria

Inclusion criteria included girls aged 10-19 years, studying in grades 6-10 at the selected school, having experienced menarche, providing informed assent with parental or guardian consent, and being willing to participate.

Exclusion criteria

Exclusion criteria included girls who have not yet experienced menarche, those with major developmental disabilities or mental health conditions impeding participation, those lacking parental or guardian consent, those declining to provide assent, and those having significant health conditions requiring intensive medical care.

Data collection and tool

Data collection was performed from March to September 2023 using a pretested, structured questionnaire adapted from past studies [12,13]. The questionnaire collected information on sociodemographic characteristics, knowledge, attitudes, and menstrual hygiene practices. It included mostly closed-ended questions with multiple-choice responses. The knowledge questionnaire comprised 15 items, with true/false and multiple-choice questions. A score of 1 was assigned for each correct response, for a total score of 15. The attitudes questionnaire comprised 10 items rated on a five-point Likert scale. Scores ranged from 1 (strongly disagree) to 5 (strongly agree), for a maximum score of 50. The practices questionnaire comprised 15 items, with frequency-based multiple-choice questions. Scores ranged from 1 to 5 depending on the frequency option, for a maximum score of 75.

The questionnaire was prepared in the vernacular language, pilot-tested, and refined before use with a Cronbach’s alpha of 0.783. The field investigators met the girls at their schools and completed the questionnaire through face-to-face interviews after obtaining written assent.

Ethical considerations

Ethical approval was obtained from the Institutional Human Ethics Committee (reference number: 207/03/2023). Written permission to conduct the study in schools was granted by the school authorities. The purpose and process of the study were explained to the participants, and written assent was obtained before the interview. Participation was purely voluntary, and confidentiality was maintained throughout.

Data analysis

Data were encoded and analyzed using SPSS version 23 statistical software (IBM Corp., Armonk, NY, USA). Descriptive statistics were calculated for sociodemographic characteristics (mean and standard deviation) while frequencies and percentages were computed for categorical variables related to menstrual hygiene knowledge, attitudes, and practices. Binary logistic regression analysis was performed to identify factors associated with good menstrual hygiene practices. Adjusted odds ratios (AORs) and 95% confidence intervals (CIs) were calculated. All statistical tests were performed at the 5% level of significance.

## Results

Table 1 presents the sociodemographic characteristics of 361 adolescent girls. The majority of participants were aged 16-19 (52.9%) years, residing predominantly in urban areas (73.4%). Father’s education levels varied, with the highest percentage having a graduate or higher education (60%), while mothers exhibited a similar trend. Nuclear families were the most common (69%), and the majority followed Hinduism (95.3%). The use of pit latrines was widespread (97.2%), and most participants lived in pucca houses (65.7%). The socioeconomic status indicated a higher representation in the upper class (56%).

**Table 1:** Sociodemographic characteristics of the participants (N = 361).

Variables	Categories	Frequency, N	Percentage
Age	10–15 years	170	47.09
	16–19 years	191	52.9
Residence	Rural	96	26.59
	Urban	265	73.4
Father’s education	Illiterate	6	1.7
	Primary/Secondary school	54	15
	Higher secondary school	86	23.8
	Graduate and higher	215	60
Mother’s education	Illiterate	23	6.4
	Primary/Secondary school	87	24.1
	Higher secondary school	75	20.8
	Graduate and higher	176	48.8
Type of family	Nuclear	249	69
	Joint	84	23.3
	Three generation	28	7.8
Religion	Hindu	344	95.3
	Others	17	4.7
Type of latrine used	Pit latrine	351	97.2
	Open defecation	10	2.8
Type of house	Pakka	237	65.7
	Hostel	115	31.9
	Semi pakka	9	2.5
Socioeconomic status	Upper (class 1 and 2)	201	56
	Lower (class 3–5)	160	44

Table 2 presents the obstetric and gynecological characteristics of 361 adolescent girls, providing valuable insights into their reproductive health. The majority experienced menarche between the ages of 12 and 15 years (81.71%), with a small percentage having an earlier onset (17.56% at <12 years) or later onset (1.11% at >15 years). Most participants reported regular menstrual cycles (87.53%), while a noteworthy proportion experienced irregularity (12.46%). A family history of dysmenorrhea was present in 11.35% of cases. Concerning the duration of menstrual flow, the study revealed that 74.79% experienced menses for three to five days, while 13.85% had a longer duration (more than five days). Pain during menstruation was reported by 65.65% of participants. Regarding the interval between menstrual cycles, the majority fell within the range of 21-38 days (86.14%), with a smaller proportion experiencing shorter or longer intervals. The frequency of changing pads per day varied, with 73.4% requiring fewer than five changes, 22.71% changing pads five to 12 times, and 3.87% exceeding 12 changes. Pre-menstrual symptoms were diverse, with 28.25% reporting a combination of nausea, body aches, and food cravings, while other participants reported symptoms such as acne, bloating, mood swings, and various combinations.

**Table 2:** Obstetric and gynecological characteristics of 361 adolescent girls.

Variables	Categories	Frequency	Percentage
Age of Menarche	<12 years	19	5.26
	12–15 years	295	81.71
	>15 years	47	13.01
Regularity of menses	Regular	316	87.53
	Irregular	45	12.46
Family history of dysmenorrhea	Yes	41	11.35
	No	320	88.64
Duration of menses flow	<3 days	41	11.35
	3–5 days	270	74.79
	>5 days	50	13.85
Pain during menstruation	Yes	237	65.65
	No	124	34.35
Interval of getting menses every monthly	<21 days	29	8.03
	21–38 days	311	86.14
	>38 days	21	5.81
Frequency of changing pads (per day)	<5	265	73.4
	5–12	82	22.71
	>12	14	3.87
Pre-menstrual symptoms (multiple answers)	Acne	32	8.86
	Acne, bloating	62	17.17
	Acne, bloating, body ache	24	6.64
	Acne, bloating, body ache, mood swings	82	22.71
	Nausea, body ache, food craving	102	28.25

As shown in Table 3, knowledge regarding menstrual bleeding among 361 adolescent girls shed light on their awareness and sources of information. A significant majority (84.21%) indicated prior knowledge about menstruation before experiencing menarche, while a notable proportion (15.79%) reported discovering this information only after the onset of menarche. Among those who were aware before menarche, the primary source of information for a substantial majority (82.54%) was their mothers, emphasizing the pivotal role of maternal guidance in imparting knowledge about menstruation. Alternatively, 17.46% of participants received information from sources other than their mothers. The overall menstrual hygiene knowledge was evenly distributed, with 47.65% having good knowledge and 52.35% having poor knowledge, emphasizing the need for targeted educational interventions.

**Table 3:** Knowledge regarding menstruation among 361 adolescent girls.

Questions	Strongly disagree (0), N (%)	Disagree (1), N (%)	Agree (2), N (%)	Strongly agree (3), N (%)
Menstruation				
1. It is a normal phenomenon	13 (3.60%)	19 (5.26%)	23 (6.37%)	306 (84.77%)
2. It is a lifelong process	41 (11.36%)	45 (12.47%)	43 (11.91%)	232 (64.27%)
3. It is a sign of conception	92 (25.49%)	73 (20.22%)	51 (14.12%)	145 (40.17%)
4. It is unique to females	4 (1.11%)	4 (1.11%)	55 (15.23%)	298 (82.55%)
5. It has a foul smell	74 (20.50%)	138 (38.23%)	85 (23.55%)	64 (17.72%)
Sources of bleeding				
1. Uterus	19 (5.26%)	21 (5.82%)	2 (0.55%)	300 (83.16%)
2. Bladder	280 (77.56%)	22 (6.10%)	9 (2.49%)	50 (13.85%)
3. Vagina	25 (6.93%)	301 (83.38%)	5 (1.39%)	30 (8.30%)
Causes of menses				
1. Hormonal	5 (1.39%)	4 (1.11%)	50 (13.85%)	302 (83.66%)
2. Disease	301 (83.38%)	30 (8.30%)	16 (4.43%)	8 (2.21%)
3. Curse	301 (83.38%)	52 (14.40%)	6 (1.66%)	2 (0.55%)

Among the key findings, a substantial 57.8% agreed that their families celebrated their menarche, reflecting positive familial responses. While 67.2% felt permitted to touch others during menstruation, there was notable disagreement (42.11%) about accessing the kitchen during this period, suggesting potential cultural restrictions. Furthermore, a significant 64.54% disagreed with discomfort discussing menstruation, indicating a level of openness. However, a prominent 52.91% agreed that activities by menstruating females are not considered blessed, revealing cultural perceptions. Attitudes toward restrictions varied, with 57.89% expressing dislike for them, while 47.65% considered being free from menses as a fate (Table 4).

**Table 4:** Attitudes regarding menstrual practices among adolescent girls (N = 361).

Questions	Strongly disagree (0), N (%)	Disagree (1), N (%)	Agree (2), N (%)	Strongly agree (3), N (%)
Your family celebrated at your menarche	17 (4.71%)	29 (8.04%)	106 (29.36%)	209 (57.89%)
You are allowed to touch others during menstruation	19 (5.27%)	31 (8.59%)	69 (19.11%)	242 (67.03%)
You are allowed to go to the kitchen during menstruation	152 (42.11%)	101 (27.98%)	22 (6.09%)	86 (23.82%)
It isn't very comfortable/ not good/ to discuss with someone about menses	20 (5.54%)	12 (3.32%)	96 (26.59%)	233 (64.55%)
Activities done by menstruating females are not blessed	22 (6.10%)	14 (3.88%)	191 (52.91%)	134 (37.12%)
You don’t like restrictions during menstruation	6 (1.66%)	123 (34.07%)	24 (6.65%)	208 (57.63%)
Being free from menses is a fate	54 (14.96%)	32 (8.87%)	173 (47.92%)	102 (28.25%)

Key findings from the menstrual hygiene practices of 361 adolescent girls highlight a predominant use of sanitary pads (96.12%), with only a minority relying on clothes (3.05%). Among cloth users, 81.81% cleaned them with water and soap. Diverse practices were observed in drying locations, with 45.44% hiding them inside the house. Reused absorbents were stored normally by 60.11%. A majority (54.3%) changed their absorbents over three times a day. Disposal methods primarily involved dustbins (79.14%). Daily bathing during menstruation was reported by 96.67%, while 60.95% cleaned external genitalia with water and soap. Overall, 39.33% exhibited good menstrual hygiene practices, emphasizing the need for targeted interventions among 60.67% with poor practices (Table 5).

**Table 5:** Menstrual practices of participants (N = 361).

Practice-related questions		Categories	Frequency	Percentage
1.	Type of absorbent used	Clothes	11	3.05
		Sanitary pads	347	96.12
		Others	3	0.83
2.	Reason for using clothes (n = 11)	I had some disbelief about them	6	54.54
		Others are not comfortable	5	45.46
3.	Cleaning of menstrual cloth (n = 11)	Don’t clean	0	0
		Only with water	2	18.19
		With water and soap	9	81.81
4.	Place of drying menstrual cloth (n = 11)	Outside in sunlight	2	18.19
		Inside the house but in the open	4	36.37
		Inside the house in hiding	5	45.44
5.	Place of keeping reused menstrual absorbent	Hidden place	144	39.89
		Normal like other clothes	217	60.11
6.	Changes of absorbent >3 times/day	Yes	196	54.3
		No	165	45.7
7.	Disposal of sanitary pads (n = 350)	Burn them	69	19.71
		Dustbin	277	79.14
		Flush in toilet	4	1.15
8.	Do you bath daily during menstruation?	Yes	349	96.67
		No	12	3.33
9.	Cleaning external genitalia	Only with water	141	39.05
		With water and soap	220	60.95
Overall menstrual hygiene practice		Good	142	39.33
		Poor	219	60.67

Table 6 presents the results of multivariate analyses examining factors associated with good menstrual hygiene practices among 361 adolescent girls. In the multivariate analysis, adjusting for other variables, age (16-19 years), higher levels of mother’s and father’s education, using pit latrines, and possessing knowledge about menstruation remained significant predictors of good menstrual hygiene practices. Specifically, adolescent girls aged 16-19 years were 2.08 times (AOR = 2.08, 95% CI = 1.14-3.81) more likely to exhibit good menstrual hygiene practices compared to those aged 10-15 years.

**Table 6:** Factors associated with good menstrual hygiene practices among 361 adolescent girls. *: p < 0.05 is significant; **: p < 0.001 is highly significant. COR = crude odds ratio; AOR = adjusted odds ratio

Variable	Category	COR	AOR
Age	10–15 years	1	1
	16–19 years	2.18 (1.5-2.90)*	2.08 (1.4-2.6)*
Area of residence	Urban	1	1
	Rural	1.03 (0.34-2.1)	1.08 (0.4-1.8)
Mother’s education	Illiterate	1	1
	Primary/Secondary school	2.006 (1.7-5.13)*	3.8 (2.5-40)*
	Higher secondary school	3.33 (1.3-8.16)**	4.05 (3.5-44)**
	Graduate and higher	4.18 (1.8-5.86)**	4.6 (3.1-50)**
Father’s education	Illiterate	1	1
	Primary/Secondary school	5.8 (1.6-53)**	2.2 (1.04-3.84)**
	Higher secondary school	8.7 (1.8-76)**	3.02 (1.62-6.28)**
	Graduate and higher	7.6 (2-68)**	4.12 (1.08-5.65)**
Family type	Nuclear	1	1
	Joint	0.7 (0.4-1.23)	0.6 (0.5-1.5)
	Three generation family	1.3 (0.5-3)	1.1 (0.6-3.2)
Type of latrine used	Open defecation	1	1
	Pit latrine	6.04 (1.7-48)**	6.7 (1.6-1.82)**
Type of house	Semi pakka	1	1
	Pakka	2.0 (1.4-7.14)*	1.129 (0.6-1.82)
	Hostel	2.17 (1.5-8.54)*	1.036 (0.22-4.8)
Knowledge	Yes	8.53 (4.5-10.4)**	8.21 (4.2-11)**
	No	1	1
Heard about menses before menarche	Yes	2.1 (1.2-3.60)	2.8 (1.4-4.80)
	No	1	1
Freely discuss with parent/family	Yes	4.3 (2.41-6.18)**	5.5 (2.5-10)**
	No	1	1

Higher levels of maternal and paternal education showed a positive association, with the odds of good menstrual hygiene practices increasing with higher educational attainment (AOR = 1.96, 95% CI = 1.04-3.71 for secondary education and AOR = 3.54, 95% CI = 1.79-7.01 for graduation and above in mothers; AOR = 2.31, 95% CI = 1.22-4.36 for secondary education and AOR = 2.86, 95% CI = 1.48-5.53 for graduate and above in fathers).

The multivariate analysis identified several crucial determinants. Age, education levels of both parents, the type of latrine used, and possessing knowledge about menstruation emerged as significant predictors. Notably, the odds of practicing good menstrual hygiene increased with higher levels of education among both mothers and fathers (as quantified above), emphasizing the pivotal role of parental education in shaping adolescent girls’ behaviors.

## Discussion

The present research delves into various facets of the reproductive health landscape among 361 adolescent girls, offering a comprehensive understanding of their sociodemographic characteristics, menstrual practices, knowledge, and attitudes. The mean age group of 15.14 ± 2.66 years in our study aligns closely with the findings of Dambhare et al. [12], where the mean age of schoolgirls was reported as 15.45 ± 1.75 years. However, there is a notable difference in the mean age observed by Shoor et al. [13], whose study reported a mean age of 13.05 years ± 0.09. This disparity could be attributed to variations in factors such as religion, socioeconomic status, and community outlook.

Regarding knowledge, our study resonates with Shanbag et al. [14], as both studies emphasize the perception of menstruation as a normal phenomenon. Shanbag et al. [14] reported that 73.7% of girls perceived menstruation as normal. In our study, a positive familial response was indicated by 58.12% celebrating the girls’ menarche. Similarly, awareness about menstruation being a unique process for females aligns with the findings of Shanbag et al. [14], who reported a percentage of 89.1%. In the present study, 82.54% said that mothers were primary informants, which is similar to the study done by Shoor et al. [13] (84.8%), but a study done by Abhay et al. [15] reported that mothers were the primary informants in only 40.67% of cases. This difference may arise because Abhay et al. [15] included school-going adolescent girls in a rural area only. The reliance on mothers as the predominant source of information suggests the potential efficacy of targeted maternal education programs aimed at enhancing reproductive health knowledge among adolescent girls. Additionally, understanding the sources of information can inform the development of comprehensive educational strategies to address the needs of those who may not have received knowledge about menstruation before its onset.

Regarding practices, Shanbag et al. [14] observed that during menstruation, 34.7% used cloth, 44.1% used sanitary pads, and 21.2% used both. Our study aligns with these findings, as the majority (96.12%) predominantly used sanitary pads. The frequency of changing sanitary pads or cloth in our study is consistent with Shanbag et al. [14] findings, where 39.8% changed twice a day, 29.5% three times a day, and 21.7% once a day. The practice of cleaning external genitalia after voiding, reported by 53.8% in the study by Shanbag et al. [14], is also echoed in our study. Promising improvements are visible, but gaps persist among subsets of girls regarding the optimal frequency of changing absorbents, genitalia cleaning, and continuing cloth reliance. Beyond quantitative data, exploring motivators and barriers around menstrual practices can explain if changes merely reflect the availability or improved agency and self-care among adolescent girls.

The prevalence of modern menstrual hygiene products, with 96.12% using sanitary pads, indicates a positive trend. However, the persistence of cloth usage in a small percentage underscores the need for continued education and awareness campaigns.

In comparing the attitudes of adolescent girls toward menstruation-related restrictions, our study reflects a positive acknowledgment of menarche through familial celebrations. Yet, disparities emerged regarding social interactions and access to spaces during menstruation, suggesting potential cultural taboos or household-specific practices that warrant exploration. The study underscores the multifaceted nature of attitudes surrounding menstruation and highlights the need for culturally sensitive interventions.

Limitations

The study’s limitations include potential recall bias, as certain information relies on participants’ memories, and the cross-sectional design, which limits the establishment of causal relationships and conducting face-to-face interviews for self-report data collection on sensitive issues around menstruation hygiene can potentially introduce social desirability and reporting biases among adolescent girls, limiting data reliability. This could be considered an important limitation of the study methodology. Alternative techniques like self-administered surveys may enhance privacy and truthful disclosure on sensitive aspects related to menstrual practices Nonetheless, the research provides a foundation for targeted interventions, emphasizing the importance of educational programs targeting both adolescent girls and their parents.

The strength of the study includes a robust sample size. The study has a sufficient sample size of 361 adolescent girls calculated using a standard statistical formula, providing adequate power. The multistage stratified random sampling technique ensured the representativeness and generalizability of results. The questionnaire was comprehensive, pretested, adapted from validated studies, and context-appropriate with mainly close-ended questions reducing subjectivity. Appropriate statistical tests were utilized including bivariate and multivariate analyses to determine associated factors.

## Conclusions

The study offers vital insights into the menstrual knowledge, attitudes, and practices among adolescent girls in Gujarat. Despite the nearly universal adoption of sanitary pad usage, gaps persist in accurate menstrual knowledge, attitudes reflect problematic sociocultural influences, and inadequate hygiene practices remain widespread. This underscores the need for multidimensional interventions encompassing health education campaigns targeting knowledge gaps among adolescent girls as well as parental engagement to shape supportive attitudes, dismantling cultural taboos through community participation, enhancing access to sanitation and private washing facilities in schools, informed policymaking, and future research. The findings can inform tailored educational programs and behavior-change communication strategies involving mothers, teachers, community leaders, and the health system. Future research could explore qualitative perceptions and barrier analysis from school and family perspectives and assess the impact of tailored interventions over time using a robust study design. Addressing menstrual health holistically remains imperative for the dignity, well-being, and empowerment of adolescent girls in India.
